# Phenotypic variability in a cohort of 40 Italian subjects carrying mutations in the gene *EDA*

**DOI:** 10.1186/1746-160X-8-S1-P2

**Published:** 2012-05-25

**Authors:** L Guazzarotti, G Tadini, GE Mancini, S Giglio, I Sani, P Nannini, M Bosoni, A Bottero, A Caimi, M Morelli, GV Zuccotti

**Affiliations:** 1 Department of Pediatrics, University of Milan, Italy; 2 IRCCS Ca' Granda Policlinico Hospital, Milan, Italy; 3 IRCCS Galeazzi, Milan, Italy; 4 University of Florence, Italy; 5 University of Milan, Italy

## 

Ectodermal dysplasias (ED) are a group of clinically and genetically heterogeneous conditions commonly characterized by abnormal development of at least two structures derived from the embryonal ectoderm (hair, teeth, nails, and sweat glands). X-linked hypohidrotic ED, which is caused by mutations in the gene *EDA* (MIM 305100), is the most frequent form. In this study, we investigated the phenotype of 40 male patients, aged 2 to 20 years, who all showed developmental defects of ectodermal derivatives and a mutation in *EDA*. Specialist assessments of the involved organ systems were performed. 95% of these patients presented with impairment of sweating, which was only moderate in 35%, while two subjects did not show any alteration of sweat gland function. Severe oligodontia was found in 80% of the subjects, 10% had hypodontia. 90% of the patients showed abnormal crown morphology of the teeth. Severe involvement of the scalp hair was observed in 22% of the patients, moderate involvement in 72%, and no relevant alterations of hair morphology, quantity or growth in 2 patients. Onychodystrophy was seen in 67% of our patients. Concerning minor alterations of ectodermal tissues, we found dry eye signs in 92% of the subjects investigated, recurrent respiratory infections in 82%, hearing loss in 10%, atopic dermatitis in 35%, and a neuropsychological disorder in 10%. Our study shows that an *EDA* mutation can be present in males also in the context of only one of the major clinical signs of X-linked ED, but associated with minor alterations. Therefore we suggest that *EDA* gene analysis should be considered also for males with a mild ED phenotype.

